# Virtual Reality-Based Cognitive–Motor Rehabilitation in Older Adults with Mild Cognitive Impairment: A Randomized Controlled Study on Motivation and Cognitive Function

**DOI:** 10.3390/healthcare8030335

**Published:** 2020-09-11

**Authors:** Ji-Su Park, Young-Jin Jung, Gihyoun Lee

**Affiliations:** 1 Advanced Human Resource Development Project Group for Health Care in Aging Friendly, Industry, Dongseo University, Busan 47011, Korea; jisu627@hanmail.net; 2 Department of Radiological Science at Health Sciences Division in DongSeo University, Busan 47011, Korea; microbme@dongseo.ac.kr; 3 Department of Physical and Rehabilitation Medicine, Center for Prevention and Rehabilitation, Heart Vascular Stroke Institute, Samsung Medical Center, Sungkyunkwan University School of Medicine, Seoul 135701, Korea

**Keywords:** virtual reality, motivation, cognitive function, mild cognitive impairment

## Abstract

The purpose of this study was to investigate the effects of virtual reality-based cognitive–motor rehabilitation (VRCMR) on the rehabilitation motivation and cognitive function in older adults. This study enrolled 40 older adults with mild cognitive impairment (MCI), living in the community. The subjects were randomly assigned to a VRCMR group (*n* = 20) or a conventional cognitive rehabilitation (CCR) group (*n* = 20). The VRCMR group underwent VRCMR using MOTOcog, a computer recognition program, whereas the CCR group underwent conventional cognitive rehabilitation, which included puzzles, wood blocks, card play, stick construction activity, and maze activity. Both interventions were performed 30 min per day, 5 days/week, for 6 weeks. This study performed a cognitive assessment using the Montreal Cognitive Assessment (MoCA) scale, Trail Making Test A and B (TMT-A/B), and Digit Span Test forward and backward (DST-forward/backward). In addition, a 0-to-10 numeric rating self-report scale was used to assess interest and motivation during the rehabilitation training. After the intervention, the VRCMR group showed a significantly greater improvement in the MoCA (*p* = 0.045), TMT-A (*p* = 0.039), TMT-B (*p* = 0.040), and DST-forward (*p* = 0.011) scores compared to the CCR group, but not in the DST-backward score (*p* = 0.424). In addition, subjects in the experimental group had significantly higher interest (*p* = 0.03) and motivation (*p* = 0.03) than those in the control group. Cohen’s d effect size was 0.4, 0.3, 0.35, 0.4, and 0.5 for the MoCA, TMT-A, TMT-B, DST-forward, and DST-backward tests, respectively. This study demonstrates that VRCMR enhances motivation for rehabilitation and cognitive function in older adults with MCI better than CCR.

## 1. Introduction

Mild cognitive impairment (MCI) has been identified as a preclinical stage of dementia and is an important predictive risk factor for dementia [1]. Although the conversion rate to dementia in patients with MCI varies depending on diagnostic criteria and evaluation tools, it is estimated at 10–15% per year [2]. This is significantly higher than the conversion rate of 1–2% in normal adults over 65 years of age [3,4]. MCI is not an obvious pathological condition but is important in terms of early intervention, in that it can be objectively or clinically determined and indicates a pathological change [5].

Attention and memory are important cognitive functions in elderly adults, which significantly decline with the development of dementia [6]. A decrease in cognitive function is related to decreased self-esteem, depression, and impaired ability to perform daily activities in elderly adults with MCI. The better the cognitive function, the higher the self-esteem, and the worse the cognitive function and self-esteem, the more severe the depression [7]. Therefore, cognitive rehabilitation therapy is important to maintain and improve cognitive function in elderly adults with MCI.

Conventional cognitive rehabilitation (CCR) is a somewhat ambiguous term that encompasses mostly tabletop activities (e.g., puzzles, wood blocks, card play, stick construction activity, and maze activity) that have long been used in rehabilitation departments to improve memory, attention, performance, and problem-solving ability of patients with cognitive impairment [8,9,10]. However, when using CCR, it is difficult not only to systematically evaluate patients’ cognitive level but also to induce interest and motivation for rehabilitation. In order to overcome these limitations, computer-based cognitive rehabilitation has been developed, and several studies have found it to be effective in improving the cognitive function of patients with neurological disorders and dementia and of older adults [11,12,13]. This therapeutic modality not only can systematically adjust the difficulties of the activities according to the cognitive level of the patient, but also has the advantage of using a variety of cognitive rehabilitation programs. Computer-based cognitive rehabilitation is fundamentally different from CCR because it is static and simple in terms of training, including simply remembering, recalling, and calculating words, numbers, and pictures.

Recently, with the advancement of IT technology, virtual reality-based cognitive rehabilitation (VRCR) has been proposed [14,15]. VR facilitates the creation of a multisensory, dynamic, interactive virtual environment with a great similarity to real life [16]. In addition, VRCR is intuitive, interesting, and fun, promoting active participation by enhancing motivation for rehabilitation [17,18]. Several studies have reported that the application of exergame increases the motivation of the elderlies [19] and helps improve the cognitive function in MCI patients [20]. Nevertheless, research on VRCR therapy is lacking, and its effects are not clear. Furthermore, it is important to introduce new methods of VRCR therapy and prove their effectiveness due to the continuous development of IT technology. Therefore, in this study, we introduce VR-based cognitive–motor rehabilitation (VRCMR) and describe its effects on the cognitive function and motivation of older adults with MCI.

## 2. Materials and Methods

### 2.1. Participants

This study enrolled 40 older adults with MCI living in the community. The inclusion criteria were as follows: diagnosed with MCI through clinical examination by a neurologist, age > 65 years, Mini-Mental State Examination score > 16 (mean score; VRCMR group = 15.1 ± 2.1, CCR group = 15.5 ± 1.9), no limitation in the upper extremity ranges of motion, fair grade on manual muscle testing of upper extremity, ability to grip objects with various forms (cylindrical, spherical, and power grip), ability to follow the study instructions, independence in daily activities, ability for adequate communication, no history of neurological disorders, including stroke, no history of visual perception deficits, and consent to participate actively. The exclusion criteria were as follows: unstable medical problems, history of psychiatric disorders, severe communication difficulties, problem with visual and auditory functions (e.g., color blindness, hearing impairment).

### 2.2. Ethics

The objectives and requirements of the study were explained to all participants, who voluntarily signed an informed consent form. Ethical approval was obtained from the Seoul medical center Institutional Review Board prior to study commencement (SEOUL2019-09-002-001).

### 2.3. Sample Size Estimation

The sample size was calculated using G-power 3.1.9.3 software (University of Dusseldorf, Dusseldorf, Germany). The power and alpha levels were set at 0.80 and 0.05, respectively, and the effective size was set at 0.9. According to a prior analysis, each group required at least 16 subjects. Therefore, 20 participants in each group were enrolled, considering possible dropouts.

### 2.4. Study Design and Procedures

**Figure 1:**
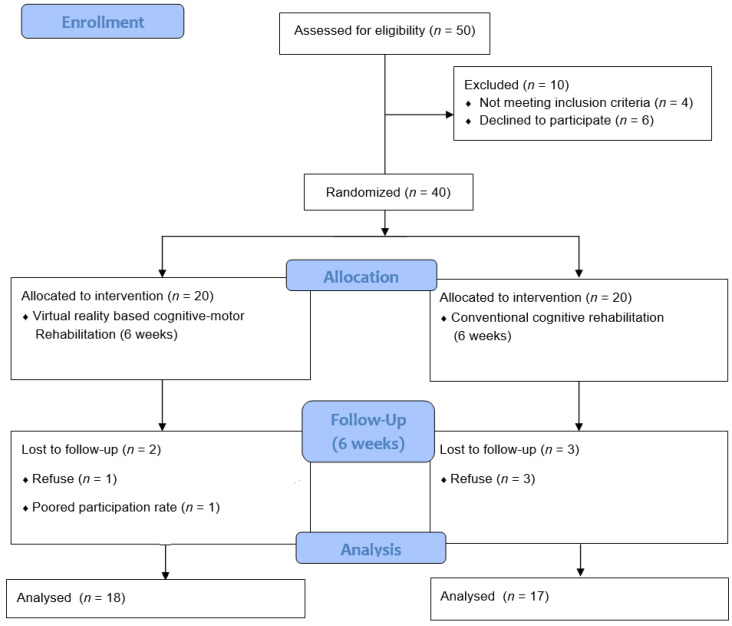
Flow diagram for this study.

The study was conducted in two groups of subjects on a 6-week schedule. Subjects were randomly assigned to either the VRCMR group (*n* = 20) or the CCR group (*n* = 20) using blocked randomization after taking baseline measures. Allocation was concealed using sealed opaque envelopes. All experimental procedures were performed by occupational therapists with more than 8 years of clinical experience. Figure 1 shows the flow diagram for this study.

**Figure 2:**
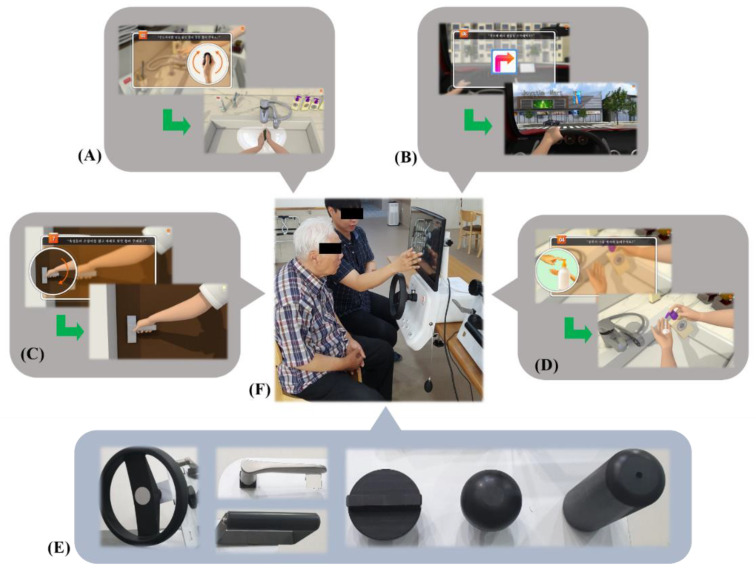
Virtual reality-based cognitive–motor rehabilitation. (**A**) After entering the bathroom, personal hygiene; (**B**) driving; (**C**) door opening; (**D**), shampoo; (**E**) attachable handles in various forms; (**F**) virtual reality-based cognitive–motor rehabilitation using Motocog.

VRCMR was performed using the MOTOCOG^®^ system (Cybermedic Inc., Gwangju, Korea). This equipment was developed specifically for the purpose of VRCMR therapy. It includes a software that uses VR for performing activities such as driving, bathing, cooking, and shopping, enabling attention, memory, problem-solving, and executive training. The hardware consists of a touchscreen monitor, grip air bulb, and various joysticks or attachments (e.g., thumb pinch, doorknob, button, gas valve, tool turn, steering wheel). Thus, preserved ranges of motion and strength of the upper limbs are required for continuous manipulation of the grip air bulb and various joysticks or attachments in relation to the realistic environment (Figure 2). VRCMR sessions were conducted 30 min per day, 5 days/week, for 6 weeks by two experienced occupational therapists.

CCR was performed with tabletop activities, including puzzles, wood blocks, card play, stick construction activity, maze and pencil–paper with table activities. The selection and level of tasks and training programs were chosen by experienced occupational therapists to match the patient’s cognitive function. CCR sessions were performed 30 min per day, 5 days/week, for 6 weeks.

### 2.5. Outcome Measurement

The following tests with proven sensitivity, validity, and reliability were used for cognitive function evaluation: Montreal Cognitive Assessment (MoCA) [21], Trail Making Test A and B (TMT-A/B) [22], and Digit Span Test forward and backward (DST-forward/backward) [23].

Evaluations were performed immediately before the start of the intervention (pre-training) and after 6 weeks of intervention (post-training). A 0 to 10 numeric rating self-report scale (NRSS) was used to assess the interest and motivation of the subjects during training [24]. The subjects completed the questionnaire after each training, and the average of all scores was calculated. Higher scores indicated higher interest and motivation for rehabilitation.

### 2.6. Statistical Analysis

All statistical analyses were performed using SPSS 15.0 software (SPSS Inc., Chicago, IL, USA). Descriptive statistics are presented as means with standard deviations. The Shapiro–Wilk test was used to check the normality of the outcome variables. To evaluate the training effects, the paired *t*-test was used to compare measures before and after the intervention in each group. The independent *t*-test was used to compare post-intervention values and changes in outcome measures between the two groups. The significance level was set at *p* < 0.05. In addition, the effect sizes (Cohen d) of the changed scores between the two groups were calculated. An effect size of 0.2, 0.5, and 0.8 represented a small, moderate, and large effect, respectively.

## 3. Results

### 3.1. Subjects’ Characteristics

A total of 40 older adults with MCI were enrolled. There were no significant differences between the groups based on general characteristics, MoCA (*p* = 0.460), TMT-A (*p* = 0.83), TMT-B (*p* = 0.35), DST-forward (*p* = 0.50), and DST-backward (*p* = 0.75) scores. Thirty-five of the 40 subjects completed the study; 5 subjects (VRCMR group [*n* = 2] and CCR group [*n* = 3]) dropped out due to refuse or poor participation rate. Therefore, 35 subjects were included in the analysis (Table 1).

### 3.2. Cognitive Function Evaluation

Based on within-group comparisons (pre-training vs. post-training), the VRCMR group showed a statistically significant improvement in the MoCA, TMT-A, TMT-B, DST-forward, and DST-backward scores (*p* < 0.001, all). In contrast, the CCR group showed statistically significant improvement in the MoCA (*p* = 0.047), DST-forward (*p* = 0.029), and DST-backward (*p* = 0.008) scores but not in the TMT-A (*p* = 0.079) and TMT-B (*p* = 0.060) scores (Table 2).

Based on the between-group post-training comparison, the VRCMR group showed greater improvement than the CCR group in the MoCA (*p* = 0.045), TMT-A (*p* = 0.039), TMT-B (*p* = 0.040), and DST-forward (*p* = 0.011) scores, but not in the DST-backward (*p* = 0.424) (Table 2).

Regarding the amount of change in both groups, significant differences were observed in the MoCA (*p* < 0.001), TMT-A (*p* < 0.01), TMT-B (*p* = 0.009), DST-forward (*p* < 0.001) scores, but not in the DST-backward (*p* = 0.468) score (Table 3). Cohen’s d effect size was 0.4, 0.3, 0.35, 0.4, and 0.5 for the MoCA, TMT-A, TMT-B, DST-forward, and DST-backward tests, respectively (Table 3).

### 3.3. Interest and Motivation Evaluation Using NRSS

**Figure 3:**
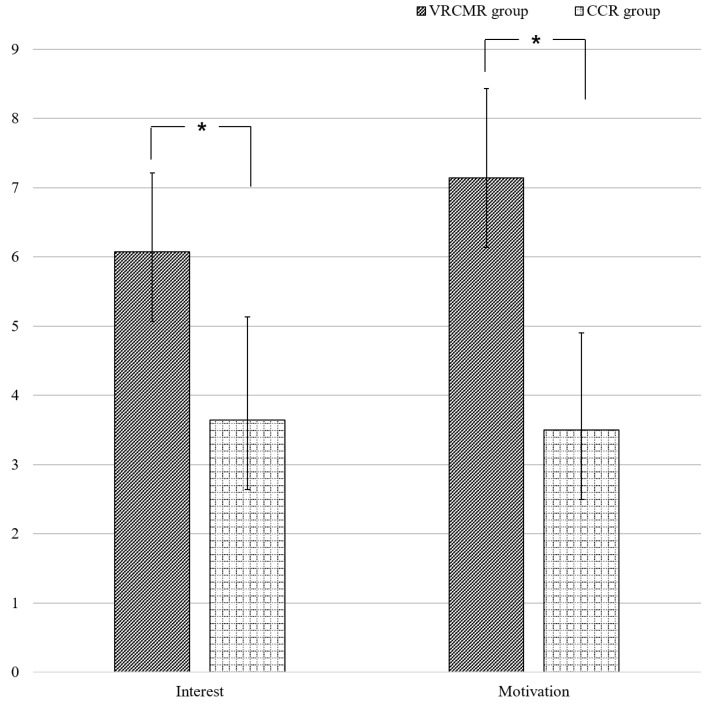
Interest and motivation evaluation using the numeric rating self-report scale. * *p* < 0.05.

The average scores for interest and rehabilitation motivation were 6.07 and 7.14 points in NRSS, respectively, for the VRCMR group, and 3.64 and 3.50 points in NRSS, respectively, for the CCR group. Based on the between-group comparison, the subjects in the VRCMR group had significantly higher interest and rehabilitation motivation than those in the CCR group (*p* < 0.001, both) (Figure 3).

## 4. Discussion

VR has been applied in various ways in the rehabilitation area, including for cognitive rehabilitation, because it has the advantage of providing fun, interest, and real-time feedback [25]. This study examined the effects of a new VRCR method, VRCMR, on the cognitive function of older adults with MCI. Our results indicate that VRCMR was more effective in improving the cognitive function of these subjects than CCR. There are several possible explanations for these findings. In this study, an NRSS was used to evaluate the subjects’ interest and motivation for training. The VRCMR group yielded significantly higher scores than the CCR group, indicating that the program offered was more fun and interesting, resulting in increased motivation for rehabilitation. In order to encourage active participation in rehabilitation, providing motivation is an important factor. This motivation theory supports the findings of our study. In particular, older adults with cognitive impairment often exhibit lethargy and depression [26]; thus, participation in rehabilitation is generally low. Because VR can increase the motivation for rehabilitation through immediate feedback, fun, and interest, it can increase training participation [18]. Increased involvement through motivation is known to trigger the thinking process by activation of brain neurotransmitter pathways, such as the cholinergic and dopaminergic systems, which helps improve concentration and memory in older people [18,27,28]. In addition, compliance is better because the surroundings are more familiar, as cognitive training is performed in virtual environments of everyday life, such as shopping, driving, cooking, and those involving calculating and opening doors [29]. Previous studies have reported that increased motivation through VR improves visual and auditory focus, which has a positive effect on the short-term visuospatial memory and attention of older adults and patients with stroke and dementia [18,27]. Therefore, in this study, higher motivation induced by through VRCMR in comparison to CCR may have helped improve the subjects’ cognitive function.

Another possible explanation for the findings of this study is brain plasticity and reorganization [30,31]. Multisensory approaches, such as VRCMR, continuously stimulate relevant brain regions. VRCMR, unlike CCR, provides continuous visual, auditory, and spatial stimuli through monitors and speakers. In VR environments, subjects are given multiple sensory modalities to interact with images and virtual objects in real time. Doniger et al. (2018) [16] reported that VRCR increased the cerebral blood flow in the prefrontal and the middle and posterior cingulate cortices, presumably due to increased brain activity during cognitive training.

Moreover, the equipment used in this study requires simultaneous continuous upper extremity movement during cognitive training. Physical exercise is known to affect cognitive functions, such as executive function, by increasing the level of brain-derived neurotrophic factor and the blood flow in the hippocampus, resulting in beneficial metabolic effects [14,16]. These outcomes might enhance the brain neuroplastic potential and enhance learning during cognitive rehabilitation [32]. In addition, physical exercise stimulates the hypothalamic–pituitary–adrenal axis, resulting in increased cortisol levels, which promote learning and memory improvement [33]. Ten Brinke et al. (2019) [13] also reported that CCR is effective in improving the cognitive function in elderly adults, but combining cognitive rehabilitation with physical exercise fosters broader benefits, similar to and supportive of the findings in this study. Therefore, both cognitive and physical exercise contribute to the process of brain repair and neuroplasticity. The findings of our study are likely due to a synergistic effect induced by performing both activities simultaneously.

This study has some limitations. First, it is difficult to generalize the results due to the small number of subjects; second, changes in brain function were not confirmed using brain imaging equipment, such as fMRI and PET; third, we did not perform follow-up evaluations after the intervention; thus, we could not determine the durability of the effects. Therefore, further large-scale studies that would include imaging evaluations and follow-up are needed to overcome the limitations of this study.

## 5. Conclusions

In conclusion, VRCMR may help improve rehabilitation motivation and cognitive function, including memory and attention, in older adults with MCI more than CCR. Therefore, VRCMR can be used as a cognitive rehabilitation intervention in older adults with MCI. However, further large-scale studies that would include imaging evaluations and follow-up are needed.

## Figures and Tables

**Table 1:** Demographic characteristics of the patients.

	VRCMR Group (*n* = 18)	CCR Group (*n* = 17)
Number of subject	18	17
Gender (man/woman)	10:8	7:10
Age (year)	75.8 ± 8.5	77.2 ± 7.2
Educational level		
Uneducated	2	1
Elementary school	13	13
Middle School	2	2
High school	1	1
University	0	0

**Table 2:** Changes in cognitive function after intervention.

	VRCMR Group (*n* = 18)			CCR Group (*n* = 17)			Between Groups *p*-Values
	Before Intervention	After Intervention	*p*-Value	Before Intervention	After Intervention	*p*-Value	
MoCA Trail Making Test	17.7 ± 3.4	20.9 ± 3.4	<0.001*	17.8 ± 2.4	18.3 ± 3.0	0.047 *	0.045 ^†^
TMT—A	72.2 ± 4.4	65.1 ± 4.4	<0.001*	69.6 ± 4.3	68.6 ± 4.6	0.079	0.039 ^†^
TMT—B Digit Span Test	152.3 ± 9.1	144.4 ± 7.7	<0.001*	154. ± 10.9	152.3 ± 11.2	0.060	0.040 ^†^
DST—forward	3.1 ± 0.8	4.7 ± 0.8	<0.001*	3.2 ± 0.8	3.7 ± 0.9	0.029 *	0.011 ^†^
DST—backward	2.0 ± 0.7	2.6 ± 0.7	<0.001*	2.0 ± 0.5	2.4 ± 0.6	0.008 *	0.424

Values are expressed as mean ± standard deviation; VRCMR: Virtual reality-based cognitive–motor rehabilitation. CCR: conventional cognitive rehabilitation. * *p* < 0.05 by paired *t* test, ^†^ *p* < 0.05 by independent *t* test.

**Table 3:** Comparison of improvement after the intervention in the two groups.

	VRCMR Group	CCR Group	*p*-Value	Cohen’s d
Δ Montreal Cognitive Assessment	3.21 ± 0.89	0.50 ± 0.85	<0.001 ^†^	0.8
Trail Making Test				
Δ Trail Making Test-A	−7.14 ± 1.70	−1.00 ± 1.96	<0.001 ^†^	0.135
Δ Trail Making Test–B	−7.93 ± 6.67	−2.14 ± 3.90	0.009 ^†^	0.6
Digit Span Test				
Δ Digit Span Test-forward	1.57 ± 0.75	0.50 ± 0.76	<0.001 ^†^	1.19
Δ Digit Span Test-backward	0.57 ± 0.51	0.43 ± 0.51	0.468	0.2

Values are expressed as mean ± standard deviation; ^†^ *p* < 0.05 by independent *t* test.
